# Case Report: Intraocular Hemorrhage in a Primary Vitreoretinal Lymphoma Patient Treated With Zanubrutinib

**DOI:** 10.3389/fmed.2022.833565

**Published:** 2022-03-22

**Authors:** Xiao Zhang, Rongping Dai, Chan Zhao, Meifen Zhang

**Affiliations:** ^1^Department of Ophthalmology, Peking Union Medical College Hospital, Peking Union Medical College, Chinese Academy of Medical Sciences, Beijing, China; ^2^Key Laboratory of Ocular Fundus Diseases, Chinese Academy of Medical Sciences and Peking Union Medical College, Beijing, China

**Keywords:** primary vitreoretinal lymphoma, intraocular hemorrhage, zanubrutinib, bruton tyrosine kinase inhibitor, intravitreal injection

## Abstract

**Purpose:**

To report a case of primary vitreoretinal lymphoma (PVRL) treated with oral zanubrutinib, who had bilateral intraocular hemorrhage after intravitreal injection of methotrexate (MTX).

**Case report:**

A 69-year-old Chinese female presented with vision decrease in both eyes. After diagnostic vitrectomy, the patient was diagnosed as PVRL in both eyes, and was treated with intravenous rituximab, oral zanubrutinib and bilateral intravitreal MTX. There were bilateral anterior chamber and vitreous hemorrhage after the fourth intravitreal MTX combined with paracentesis. After discontinuation of zanubrutinib, vitrectomy and silicon oil tamponade were performed on the left eye, and the blood in the right eye was absorbed.

**Conclusion:**

Bleeding is a major concern in the use of zanubrutinib. It is suggested that drugs be held for a few days prior to procedures and surgeries.

## Introduction

As the most common intraocular lymphoma, primary vitreoretinal lymphoma (PVRL) belongs to a rare subset of primary central nervous system lymphoma (PCNSL), and most PVRL are B-cell lymphomas (1, 2). For the treatment of B-cell malignancies, bruton tyrosine kinase (BTK) inhibitor is broadly approved owing to its sustained BTK occupancy (3). As a novel BTK inhibitor, zanubrutinib exhibits less off target inhibition than ibrutinib, which is a first-generation BTK inhibitor (4). However, compared with standard chemotherapy, BTK inhibitor has side effects of clinically significant hemorrhage. Here, we report a case of PVRL with oral zanubrutinib, who developed bilateral intraocular hemorrhage after intravitreal injection of methotrexate (MTX) and paracentesis.

## Case Report

A 69-year-old Chinese female presented to Peking Union Medical College Hospital (PUMCH) in the end of Oct 2020, complaining decreased vision in her left eye for 5 months and blurred vision with floaters in her right eye for 1 month. Her past medical history included bilateral phacoemulsification and intraocular lens (IOL) implantation performed in 2016. On presentation to PUMCH, her best correct visual acuity (BCVA) was 0.6 OD and NLP OS. Intraocular pressure (IOP) was 13 mmHg OU. In the right eye, there was no obvious anterior chamber inflammation, and there were mild dusty opacity in the vitreous and punctate gray-white lesions in the retina. In the left eye, there were gray-white keratic precipitates (KPs), mild anterior chamber cells and flare, a large amount of white granular opacity in the vitreous and blurred fundus. B-scan ultrasonography showed mild vitreous opacities in the right eye and severe vitreous opacities with proliferative membrane, as well as detachment of retina and choroid in the left eye. The results of systemic examinations including head enhanced magnetic resonance imaging were normal.

Anterior chamber paracentesis was performed in both eyes. Aqueous humor Interleukin (IL)-10 and IL-6 levels were 251.2 and 56.7 pg/mL (ratio 4.4) OD, 858.4 and 499.2 pg/mL (ratio 1.7) OS. Diagnostic vitrectomy was performed in the left eye on November 9, 2020, very few atypical cells were found in the vitreous, but gene rearrangement and flow cytometry of vitreous were all negative. As the vitreous opacity deteriorated and the vision decreased quickly to 0.1, vitrectomy was performed in the right eye on December 7, 2020, which demonstrated the monoclonality of B cell for lymphoma cells in the flow cytometry test. Meanwhile, in the cerebral spinal fluid, the IL-10 level raised significantly (108.0 pg/mL) and myeloid differentiation factor 88 (*MYD88*^L265P^) mutation was detected. Accordingly, the patient was diagnosed as PVRL OU. The treatment regimen was intravenous rituximab 600 mg, once every 21 days for a total of 6 times, combined with oral zanubrutinib 160 mg twice a day. Local treatment was bilateral intravitreal MTX for 1 year (once a week for 4 weeks, once every 2 weeks for 4 weeks, once a month for 10 months, 16 times in total).

**FIGURE 1:**
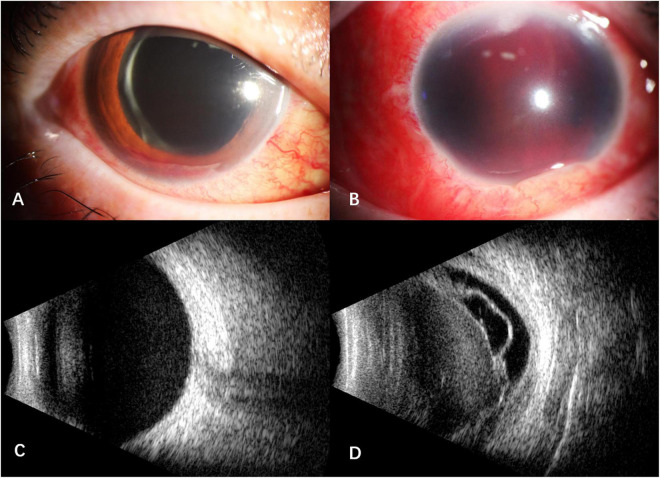
Anterior segment and B -scan ultrasonography of the patient after the fourth intravitreal MTX combined with paracentesis. **(A)** floating blood cells and a small amount of hyphema in the anterior chamber of the right eye; **(B)** the anterior chamber was full of blood in the left eye; **(C)** suspected vitreous hemorrhage in the right eye; **(D)** suspected vitreous hemorrhage with retinal detachment and proliferative membrane in the left eye.

**FIGURE 2:**
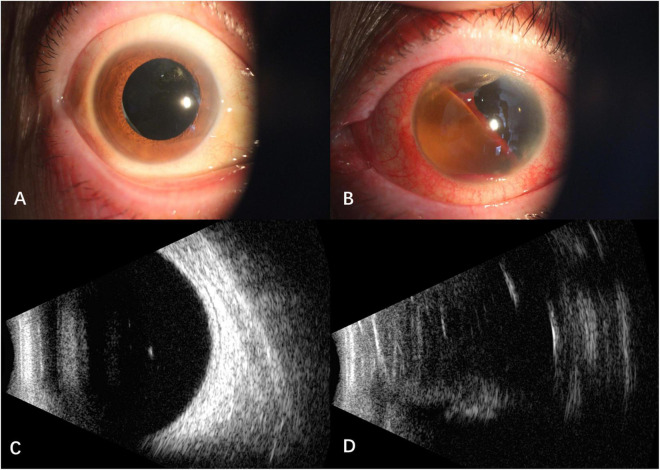
Anterior segment and B -scan ultrasonography of the patient 5 weeks after the onset of intraocular hemorrhage. **(A)** Hemorrhage in the anterior chamber of the right eye absorbed; **(B)** intraocular lens was removed and the hemorrhage absorbed in the left eye; **(C)** no vitreous hemorrhage in the right eye; **(D)** vitreous silicone oil filling with retinal detachment in the left eye.

After intravitreal MTX once a week for three times, the BCVA was 1.0 OD, NLP OS. The fourth intravitreal MTX combined with paracentesis was performed on January 12, 2021, but the patient felt blurred vision in the right eye and redness, swelling and pain in the left eye a few hours later. When the patient presented, the vision of the right eye was hand motion, IOP was normal OU. Floating blood cells and a small amount of hyphema in the anterior chamber (Figure 1A), moderate vitreous hemorrhage, and blurred fundus were found in the right eye, while the anterior chamber was full of blood in the left eye (Figure 1B). The B-scan ultrasonography showed suspected vitreous hemorrhage in both eyes (Figures 1C,D). The aqueous humor IL-10 levels were 2.59 pg/mL OD and 25.23pg/mL OS, which were significantly lower than before treatment. The blood routine and coagulation function were within the normal range, but the result of collagen-induced platelet aggregation test was 10%, which was much lower than normal (70∼94%). We then discontinued the use of zanubrutinib. Three days later, the result of collagen-induced platelet aggregation test increased to 20%, and then increased to 78% after another 4 days. The vitrectomy and silicon oil tamponade were performed in the left eye on January 22, 2021. After cleaning up the large amount of blood in the anterior chamber, we found that the IOL was dislocated to the anterior chamber. After the removal of IOL, the patient’s eye pain was greatly relieved, which may be caused by the stopping of IOL stimulation on the anterior chamber angle and the iris. When the patient presented 5 weeks after the onset of intraocular hemorrhage, the vision of her right eye increased to 0.6, IOP was 16 mmHg OD and 7 mmHg OS. Conjunctival congestion was found in her left eye, and the hemorrhage in both eyes was absorbed. The B- ultrasonography showed silicone oil filling and retinal detachment in the left eye (Figures 2A–D). She continued the intravitreal MTX injection in the right eye and the systemic treatment. Zanubrutinib was stopped 3 days before the intravitreal injection and was continued 1 day after the injection. Three months later, the BCVA of the right eye increased to 1.0.

## Discussion

Approximately 56–90% of patients with PVRL have or eventually develop central nervous system disease (1). Although the amount of PVRL patients is gradually increasing, there are no clinical guidelines for diagnosis, monitoring, and therapy until recently (2). According to the immunologic and histologic features, most PVRLs belong to the diffuse large B-cell (DLBCL) histologic subtype (1, 2).

BTK is an important component that interconnects antigen-dependent B cell receptor (BCR) signaling, toll-like receptor (TLR) signaling, and chemokine receptor signaling. These signaling pathways regulate the proliferation, survival, adhesion, and migration of B cells (3). Therefore, BTK inhibitors are good candidates for the treatment of B-cell malignancies without chemotherapy, including non-Hodgkin lymphomas and chronic lymphocytic leukemia (3).

However, the risk of bleeding should be considered in the use of BTK inhibitors. Major bleeding is less common but potentially more serious, especially in patients with concurrent antiplatelet drugs or anticoagulants (5). Such cases have been reported in the literature, e.g., central nervous system hemorrhage, severe hematuria, post-operative and post-traumatic hemorrhage, and gastrointestinal hemorrhage in colon cancer (6).

Ibrutinib is the first selective BTK inhibitor approved by US FDA (3, 7). From the meta-analysis of published trials, the overall bleeding incidence increased in patients treated with ibrutinib compared with patients in control groups, but no significant difference was found in the incidence of major bleeding (8). Another report on the risk of bleeding with ibrutinib from 15 clinical studies (*N* = 1,768) identified low-grade bleeding in 36% and major bleeding in 4.1% of patients, and consequently 1% of patients discontinued ibrutinib (9).

Zanubrutinib is a second-generation BTK inhibitor, which was approved by the US FDA to treat relapsed/refractory Mantle-Cell Lymphoma (MCL) in 2019 (3, 7). Compared with ibrutinib, it has higher BTK selectivity and exhibits less off-target inhibition of other tyrosine kinases (7, 10). In two phase II studies of zanubrutinib, major bleeding was reported in 2 out of 85 patients (2.3%), and 2 out of 91 patients (2.2%) patients, respectively (11, 12). A safety analysis from 6 zanubrutinib monotherapy trials showed that grade ≥ 3 bleeding occurred in 2.1% of 682 patients (5). Compared with ibrutinib, the rate of major bleeding with zanubrutinib is lower (13), which is supported by a phase III study (ASPEN study) (4).

The pathophysiology of hemorrhage associated with BTK inhibitors is related to the platelet function, but is irrelevant with the number of platelets. The platelet activation and thrombus stability are dependent of the platelet transmembrane receptors, whose downstream signal molecules include BTK and tyrosine kinase expressed in hepatocellular carcinoma (TEC) (6, 14). The BTK inhibitor may also inhibit TEC, and the inhibition significantly decreased the collagen receptor glycoprotein VI (GPVI) -mediated platelet activation, spreading, and aggregation *in vitro* (15, 16). In addition, the BTK inhibitor inhibits downstream signals of C-type lectin-like receptor 2 (CLEC-2), which is a platelet transmembrane receptor dependent on BTK and TEC signaling (6, 14). Besides the GPVI and CLEC-2-mediated pathways, the BTK inhibitor also interferes with the platelet glycoprotein Ib (GPIb) -mediated platelet functions, and inhibits platelet adhesion to fibrinogen (6, 15).

*In vitro* studies demonstrated that ibrutinib inhibits collagen-induced platelet aggregation and von Willebrand factor (vWF) -mediated platelet activation (14). After discontinuing ibrutinib, the collagen induced platelet aggregation partially recovered after 2.5 days and returned to normal 1 week later (6, 14). Our case developed bilateral intraocular hemorrhage after intravitreal injection, which we think is related to the application of zanubrutinib. The collagen-induced platelet aggregation test increased from 10 to 78% one week after discontinuation of zanubrutinib. To our knowledge, this is the first reported case of intraocular hemorrhage after intravitreal injection in patient with zanubrutinib treatment.

To prevent perioperative bleeding events for patients using BTK inhibitors, it is suggested holding drugs for 3 days prior to minor procedures and 7 days prior to major surgeries, with re-initiation 1–3 days later (6, 17). Meanwhile, attention should be paid to the risk of bleeding in patients with antiplatelet agents and anticoagulants (17).

## Data Availability Statement

The original contributions presented in the study are included in the article/supplementary material, further inquiries can be directed to the corresponding author/s.

## Ethics Statement

Written informed consent was obtained from the individual(s) for the publication of any potentially identifiable images or data included in this article.

## Author Contributions

XZ, RD, CZ, and MZ participated in the diagnosis and treatment of the patient. XZ wrote the manuscript. RD, CZ, and MZ carefully reviewed and revised the manuscript. All authors contributed to the article and approved the submitted version.

## Conflict of Interest

The authors declare that the research was conducted in the absence of any commercial or financial relationships that could be construed as a potential conflict of interest.

## Publisher’s Note

All claims expressed in this article are solely those of the authors and do not necessarily represent those of their affiliated organizations, or those of the publisher, the editors and the reviewers. Any product that may be evaluated in this article, or claim that may be made by its manufacturer, is not guaranteed or endorsed by the publisher.
